# Local knowledge, use, and conservation of wild birds in the semi-arid region of Paraíba state, northeastern Brazil

**DOI:** 10.1186/s13002-018-0276-x

**Published:** 2018-12-04

**Authors:** Vanessa Moura dos Santos Soares, Hyago Keslley de Lucena Soares, Suellen da Silva Santos, Reinaldo Farias Paiva de Lucena

**Affiliations:** Laboratório de Etnobiologia e Ciências Ambientais, Departamento de Sistemática e Ecologia, da Universidade Federal da Paraíba, Campus I, João Pessoa, Paraíba CEP: 58.051-900 Brazil

## Abstract

**Background:**

The use of wild birds, for several purposes, is directly associated with cultural, ecological, and conservation issues. This study aimed to inventory the wild birds known and used in three communities in Paraíba state, northeast Brazil, and to investigate the sociocultural context in which these activities occur.

**Methods:**

A total of 179 people (98 women and 81 men) were interviewed. Data were collected through free interviews, using semi-structured forms, and posing questions about the use of local wild birds. The species were identified by direct observation of the birds, analysis of photographic records, and the use of a scientific guide.

**Results:**

Each species’ use value (UV) was calculated in three different ways: UV_general_, UV_current_, and UV_potential_. These UVs ranged from 0.01 to 1.15 for UV_g_, 0 to 0.21 for UV_c_, and 0.01 to 1.02 for UV_p_. A total of 99 species, 81 genera, and 40 families were recorded and classified into the use categories of food, breeding, and medicinal. Thraupidae (12 species), Columbidae, Accipitridae, and Icteridae (8 species each) were the most diverse families.

**Conclusions:**

The use of wild birds is a widespread activity in the studied areas, where many species are used. This demonstrates the need to conduct studies to assess the pressure suffered by these bird species, as well as the need to create public policies that intervene in the use and conservation of wild birds.

## Background

South America is considered the birds’ continent because it encompasses about one third of all bird species on Earth [1]. The Brazilian Committee of Ornithological Records [2] states that 1919 species have been recorded in Brazil, 270 of which are endemic. Brazil, along with Colombia and Peru, it represents the group of the richest countries in bird diversity in the wolrd [1, 3, 4].

There are several biomes and phytogeographical regions in Brazil; Ab’ saber [5] states that Caatinga is one of the six great landscape and macroecological domains in the country. Located in the interior of northeastern Brazil, in terms of biodiversity, Caatinga is considered one of the richest semi-arid regions in the world [6] and is known as an important endemic centre for South American birds [1, 7–10]. Currently, a total of 511 species have been recorded in the Caatinga biome; this number corresponds to 30% of all birds recorded in Brazil. Currently, Caatinga has 20 endangered species and 15 endemic species [11].

Among the vertebrates, birds are the easiest group to identify, since they are active and easily seen during the day and clearly distinguish themselves from other animals due to their plumage and their ability to fly and sing [12, 13]. This has also perhaps made them vulnerable and easy targets, mainly for the illegal trade, as well as largely recognised by human populations.

In Brazil, the use of wild birds is a widespread practice in both rural and urban areas [12–14]. In both big and small cities, birds are used for different purposes and are of great social, economic, and cultural importance. Some birds are used by the local population as a source of food (meat, eggs, and bones), medicine (traditional medicine), and ornamental products (eggs and feathers), as well as being used for companionship and ornamentation (songbirds, pets) [13–15].

Birds have been present in all cultural levels of human life, from prehistory to present times [15], as components of the wild fauna and constituents of an essential and significant part of human daily life, establishing cognitive, emotional, and behavioural bonds [14, 15]. The study of these bonds and the relationships between people and birds is approached using the principles of ethnobiology, and more specifically by ethno-ornithology [14, 15], which studies the knowledge, symbolism, meaning, and attributes of birds by human communities. These people-bird relationships became important in the Brazilian semi-arid region, where edaphic and climatic factors and socioeconomic conditions led the local populations to develop a very singular sociocultural structure and a strong relationship with the faunal and floristic resources of their region [13–17].

Given the importance of wild fauna to the populations of the semi-arid region of northeastern Brazil, this study recorded and evaluated how local populations use these resources. The goal was to provide information to support the development of strategies for reducing negative impacts on the species that are used by the population, such as the development of sustainable management plans for species that are under a high pressure of use, in order to avoid their local extinction.

## Materials and methods

### Study area

**Fig. 1:**
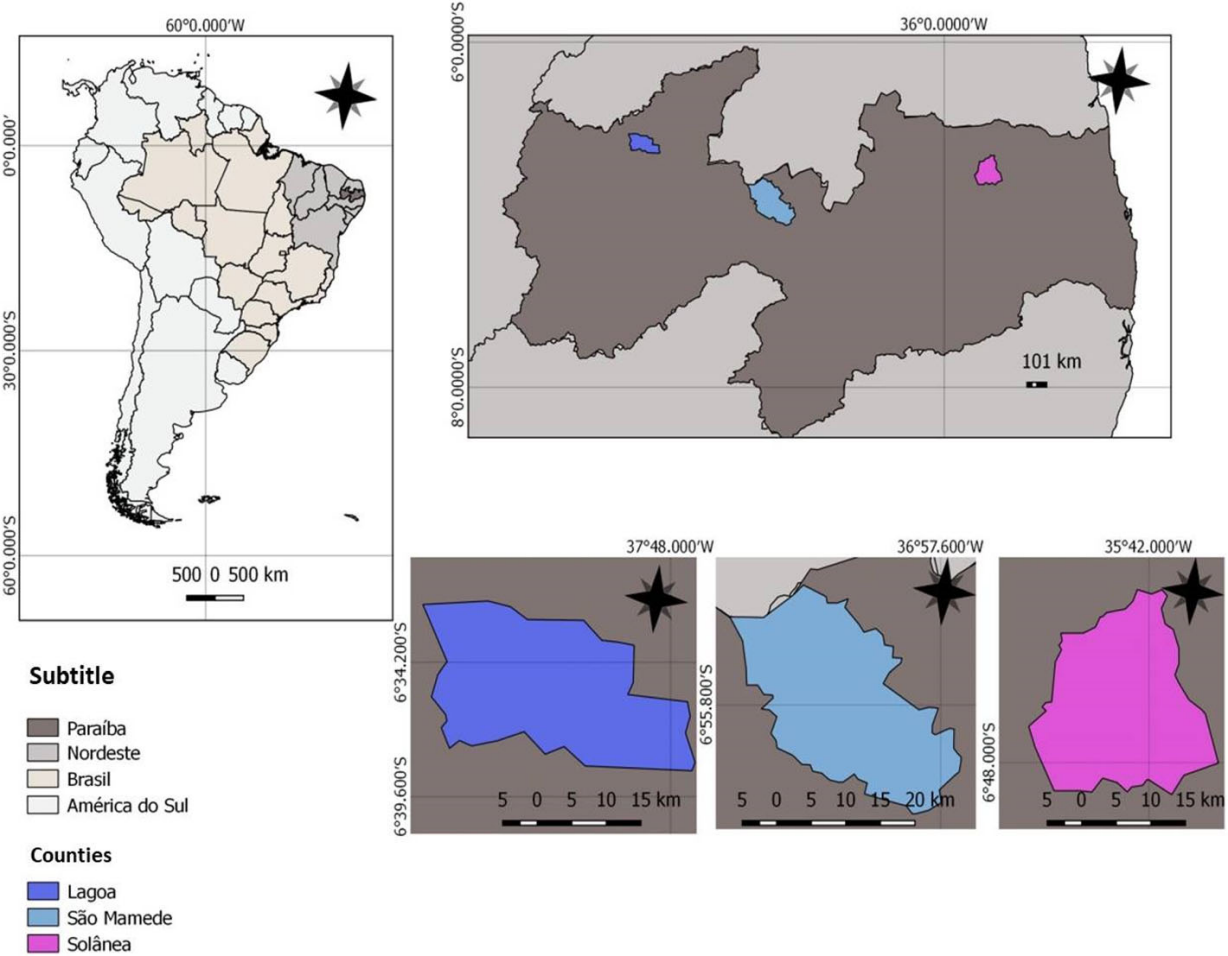
Geographic location of the study areas: Lagoa, São Mamede, and Solânea (Paraíba State, Northeast Brazil)

This study was conducted in the semi-arid region of Paraíba state, in the municipalities of Solânea, Lagoa, and São Mamede (Fig. 1). It aimed to record the residents’ knowledge and use of the birds in each region. The rural communities of Pereiro (Lagoa), Capivara (Solânea), Gatos, and Várzea Alegre (São Mamede) were visited.

The municipality of Lagoa is located in the mesoregion of Sertão (a Brazilian region with a semi-arid climate and vegetation) and in the microregion of Catolé do Rocha. It is 394 km away from João Pessoa, the state capital, at the coordinates 6° 35′ 26″ S latitude and 37° 54′ 52″ W longitude, and borders the municipalities of Bom Sucesso, Jericho, and Mato Grosso (to the north); Pombal (to the south); Paulista (to the east); and Santa Cruz (to the west). Its population of 4681 inhabitants (2010—data from the last population census conducted in Brazil) is distributed over an area of 177,901 km^2^ [18]. In Lagoa, the study was conducted in the community of Pereiro, which is approximately 1 km from the downtown area of the municipality. This community’s economy is based on agriculture, particularly sheep, goats, and cattle breeding, as well as the cultivation of beans, cotton, tobacco, and corn [18].

The municipality of São Mamede (06° 55′ 37″ S latitude and 37° 05′ 45″ W longitude) is located in the mesoregion of Borborema and the microregion of Seridó Ocidental, 283 km from the state capital, João Pessoa. It has a population of 7748 inhabitants (2010—data from the last population census conducted in Brazil) and a territorial area of 530,724 km^2^. It is bordered by Ipueira and Várzea to the north, Areia de Baraúnas and Passagem to the south, Santa Luzia to the east, and Patos and São José de Espinharas to the west. In São Mamede, the study was conducted in the communities of Gatos and Várzea Alegre, which are approximately 8 km from the downtown areas of the municipality. Its economy is based on rainfed agriculture (agriculture production occurs only during the rainy season) and consists mainly of bean and corn crops [18].

The municipality of Solânea (6° 46′ 40″ S latitude and 35° 41′ 49″ W longitude) is located in the mesoregion of Agreste and the microregion of Curimataú Oriental, 130 km away from the state capital. It is bordered by Cacimba de Dentro (north), Arara and Serraria (south), Dona Inês and Bananeiras (east), and Casserengue and Remígio (west). It has a population of 30,598 (2010—data from the last population census conducted in Brazil), distributed over an area of 265,921 km^2^ [18]. In Solânea, the study was conducted in the community of Capivara, located about 20 km from the downtown area of the municipality. It has a subsistence economy mainly based on subsistence agriculture (bean and corn) [18].

### Ethno-ornithological data collection

Ethno-ornithological data were collected through semi-structured forms, free interviews, and guided tours with some informants. The avifauna was identified through (1) direct observation of the species, (2) a scientific guide (Avis Brasilis), and (3) analysis of photographic records taken during interviews and guided tours [19], and complemented by informal conversations. The nomenclature used in this study follows the taxonomy suggested by the Brazilian Ornithological Record Committee [2]. Before each interview, a conversation was held with the informants to explain the purpose of the work, during which they were invited to sign a free and clarified consent form, required by the National Health Council through the Research Ethics Committee (Resolution 196/96). The present study was approved by the Human Research Ethics Committee (CEP) of the Lauro Wanderley Hospital, at the Federal University of Paraíba (protocol CEP/HULW No. 297/11, cover sheet No. 420/134).

The research was conducted from June 2011 to June 2012. The interviews were conducted with the two breadwinners of each family (male and female) in each community to record and evaluate their knowledge, with a total of 181 informants (98 women and 81 men). Out of these, 5 men and 17 women were from Lagoa, 26 men and 26 women were from São Mamede, and 51 men and 56 women were from Solânea. The difference between genders was due to the presence of widows and unmarried men. The ages ranged from 15 to 75 years in Lagoa, 21 to 81 years in São Mamede, and 19 to 81 years in Solânea. The semi-structured form used in the interviews included questions about the avifauna present found in the region, such as the purpose and the use attributed to these birds, methods of capturing the animals, and morphological and ecological descriptions for each species.

### Data analysis

#### Use value

The use value (UV) index was used to infer the local importance of the species, based on information from local residents. In the present study, the UV was calculated based on the proposal by Lucena et al. [20], which considers three different ways of collecting and interpreting data from interviews. Thus, the current use value (UV_c_) is based on the citations that people reported as corresponding to effective use; the potential use value (UV_p_) is based on the uses that people cited, but do not perform; and the general use value (UV_g_), which is commonly used in the literature, does not distinguish between use and knowledge.

$$ {\mathrm{UV}}_c=\frac{U_{\mathrm{ic}}}{n} $$

where UV_*c*_ = current use value of the species, *U*_*i*_ = number of citations of current use of the species mentioned by each informant, and *n* = total number of informants.

$$ {\mathrm{VU}}_{\mathrm{p}}=\frac{U_{\mathrm{ip}}}{n} $$

where UV_p_ = potential use value of the species, *U*_*i*_ = number of citations of potential use of the species mentioned by each informant, and *n* = total number of informants.

$$ {\mathrm{UV}}_{\mathrm{g}}=\frac{U_i}{n} $$

where UV_g_ = use value of the species, *U*_*i*_ = number of use citations of the species mentioned by each informant, *n* = total number of informants.

## Results and discussion

**Fig. 2:**
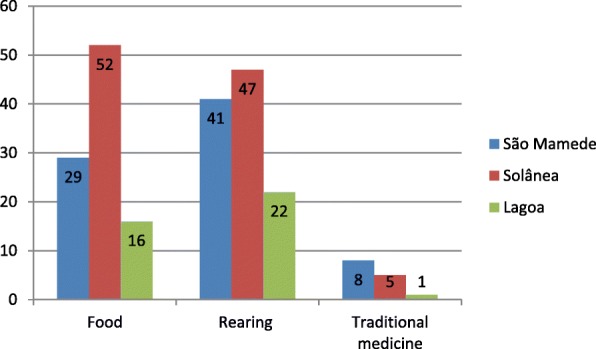
Number of species per category in the municipalities of Lagoa, São Mamede, and Solânea in Paraíba State, Northeast Brazil

A total of 99 wild bird species were recorded, distributed across 40 families and 81 genera (Table 1). These birds were used as food, raised as pets, and used for traditional medicine (Fig. 2). In the three study areas, Thraupidae (12 species), Columbidae, Accipitridae, Icteridae (8 species each), and Tinamidae (6 species) were the most diverse families. This corroborates with other studies [13–15, 21, 22] that show that the use of birds is a widespread activity in the semi-arid region of Brazil, where it is rooted in popular culture and includes several species [14, 21]. The same bird can be captured and used in several ways, along with its by-products, by several populations in Brazil and in the world [15, 17, 21, 23]. All the recorded species occur in Brazil [1, 2], and *Sporophila albogularis*, *Icterus jamacaii*, *Paroaria dominicana*, *Eupsittula cactorum*, *Cyanocorax cyanopogon*, and *Compsothraupis loricata* are characteristic species from Caatinga, a typical biome of the semi-arid region of Brazil [2, 24, 25]. *Spinus yarrellii* is classified as “vulnerable” in lists of endangered birds; populations are in decline mainly due to over-exploitation and illegal trade [12, 26].

**Table 1:** Species of wild birds used by the inhabitants in the rural communities of São Mamede, Lagoa, and Solânea, Northeast Brazil, with their general, current, and potential use values (UV)

Taxon	São Mamede-PB			Lagoa-PB			Solânea-PB			Use
	VU_g_	VU_p__p_	VU_at_	VU_g_	VU_p_	VU_at_	VU_g_	VU_p_	VU_at_	
Passeriformes										
Icteridae										
*Agelaioides fringillarius* (Spix 1824)	0.36	0.36	0							Al, Cr
*Gnorimopsar chopi* (Vieillot, 1819)	0.2	0.18	0.02	0.15	0.1	0.05	0.04	0.04	0	Al, Cr
*Icterus cayanensis* (Linnaeus, 1766)	0.18	0.18	0	0.2	0.2	0	0.04	0.04	0	Cr
*Icterus jamacaii* (Gmelin, 1788)	0.92	0.88	0.04	0.35	0.35	0	0.27	0.27	0	Al, Cr
*Procacicus solitarius* (Vieillot, 1816)	0.02	0.02	0	0.1	0.1	0				Cr
*Molothrus bonariensis* (Gmelin, 1789)	0.06	0.06	0	0.1	0.1	0	0.02	0.02	0	Cr
*Sturnella superciliaris* (Bonaparte, 1850)							0.01	0.01	0	Cr
*Icterus pyrrhopterus*(Vieillot, 1819)	0.06	0.06	0	0.05	0.05	0				Cr
Cardinalidae										
*Cyanoloxia brissonii* (Lichtenstein, 1823)	0.08	0.08	0	0.15	0.1	0.05	0.4	0.4	0	Al, Cr
Fringillidae										
*Euphonia chlorotica* (Linnaeus, 1766)	0.27	0.27	0	0.1	0.1	0	0.14	0.14	0	Al, Cr
*Spinus yarrellii* (Audubon, 1839)	0.08	0.08	0				0.13	0.13	0	Cr
Furnariidae										
*Pseudoseisura cristata* (Spix, 1824)	0.42	0.42	0	0.15	0.15	0				Cr
*Furnarius leucopus* Swainson, 1838	0.04	0.04	0	0.05	0.05	0				Cr
Corvidae										
*Cyanocorax cyanopogon* (Wied, 1821)	0.79	0.79	0	0.3	0.3	0	0.08	0.08	0	Al, Cr, Me
Thraupidae										
*Coryphospingus pileatus* (Wied, 1821)	0.08	0.08	0	0.1	0.1	0	0.13	0.12	0.01	Cr
*Tachyphonus rufus* (Boddaert, 1783)							0.01	0.01	0	Cr
*Tangara sayaca* (Linnaeus, 1766)							0.07	0.05	0.02	
*Paroaria dominicana* (Linnaeus, 1758)	1	0.94	0.06	0.8	0.8	0	1.09	1.02	0.07	Al, Cr, Me
*Sporophila albogularis* (Spix, 1825)	0.48	0.42	0.06	0.25	0.2	0.05	1.15	0.94	0.21	Al, Cr
*Sporophila angolensis* (Linnaeus, 1766)	0.02	0.02	0				0.01	0.01	0	Cr
*Sporophila bouvreuil* (Statius Muller, 1776)	0.1	0.1	0	0.05	0.05	0	0.02	0.02	0	Cr
*Sporophila lineola* (Linnaeus, 1758)	0.1	0.1	0	0.15	0.1	0.05	0.77	0.59	0.18	Al, Cr
*Sporophila nigricollis* (Vieillot, 1823)	0.04	0.04	0				0.01	0.01	0	Cr
*Sporophila leucoptera* (Vieillot, 1817)							0.01	0.01	0	Cr
*Sicalis flaveola* (Linnaeus, 1766)	0.37	0.37	0	0.15	0.15	0	0.2	0.2	0	Al, Cr
*Volatinia jacarina* (Linnaeus, 1766)							0.09	0.08	0.01	Al Cr
Mimidae										
*Mimus saturninus* (Lichtenstein, 1823)	0.54	0.52	0.02	0.25	0.25	0	0.13	0.13	0	Al, Cr
Passeridae										
*Passer domesticus* (Linnaeus, 1758)	0.12	0.12	0	0.05	0.05	0				Cr
Tyrannidae										
*Fluvicola nengeta* (Linnaeus, 1766)							0.06	0.06	0	Al, Cr, Me
*Xolmis dominicanus* (Vieillot, 1823)	0.12	0.12	0							Cr
*Pitangus sulphuratus* (Linnaeus, 1766)	0.48	0.48	0	0.15	0.15	0	0.16	0.16	0	Al, Cr
Polioptilidae										
*Polioptila plumbea* (Gmelin, 1788)	0.17	0.17	0				0.04	0.04	0	Al, Cr
Hirundinidae										
*Progne chalybea* (Gmelin, 1789)	0.06	0.06	0							Cr
Passerellidae										
*Ammodramus humeralis* (Bosc, 1792)							0.02	0.02	0	Cr
*Zonotrichia capensis* (Statius Muller, 1776)	0.04	0.04	0				0.03	0.03	0	Al, Cr
Troglodytidae										
*Troglodytes musculus* Naumann, 1823	0.36	0.36	0	0.25	0.25	0	0.04	0.04	0	Al, Cr
Turdidae										
*Turdus amaurochalinus* Cabanis, 1850	0.02	0.02	0							Cr
*Turdus leucomelas* Vieillot, 1818	0.02	0.02	0	0.3	0.3	0	0.24	0.23	0.01	Al, Cr
*Turdus rufiventris* Vieillot, 1818	0.33	0.33	0	0.35	0.35	0	0.32	0.31	0.01	Al, Cr
Thamnophilidae										
*Herpsilochmus atricapillus* Pelzeln, 1868							0.14	0.14	0	Al, Cr
*Thamnophilus capistratus* Lesson, 1840							0.01	0.01	0	Cr
Gruiformes										
Rallidae										
*Aramides cajaneus* (Statius Muller, 1776)	0.12	0.12	0	0.1	0.1	0				Al
*Gallinula galeata* (Lichtenstein,1818)	0.12	0.12	0							Al
Aramidae										
*Aramus guarauna* (Linnaeus, 1766)	0.08	0.08	0							Al
Psittaciformes										
Psittacidae										
*Eupsittula cactorum* (Kuhl, 1820)	0.52	0.52	0	0.65	0.6	0.05				Al, Cr
*Amazona aestiva* (Linnaeus, 1758)	0.04	0.04	0							Cr
*Forpus xanthopterygius* (Spix, 1824)	0.15	0.15	0				0.15	0.15	0	Cr
Pelecaniformes										
Ardeidae										
*Ardea alba* Linnaeus, 1758	0.12	0.12	0				0.02	0.02	0	Al
*Egretta thula* (Molina, 1782)	0.02	0.02	0	0.1	0.1	0				Al
*Tigrisoma lineatum* (Boddaert, 1783)	0.02	0.02	0							Al
Strigiformes										
Strigidae										
*Athene cunicularia* (Molina, 1782)	0.06	0.06	0	0.15	0.15	0				–
*Megascops choliba* (Vieillot, 1817)	0.02	0.02	0							Cr
*Glaucidium brasilianum* (Gmelin, 1788)	0.29	0.29	0	0.1	0.1	0	0.03	0.03	0	Cr
Tytonidae										
*Tyto furcata* (Temminck, 1827)	0.14	0.14	0	0.05	0.05	0	0.04	0.04		–
Accipitriformes										
Accipitridae										
*Rupornis magnirostris* (Gmelin, 1788)	0.31	0.31	0	0.1	0.1	0	0.05	0.05	0	Al, Cr
*Buteo brachyurus* Vieillot, 1816	0.1	0.1	0							Al
*Buteo nitidus* (Latham, 1790)	0.02	0.02	0							Al
*Buteo swainsoni* Bonaparte, 1838	0.02	0.02	0							Al
*Rostrhamus sociabilis* (Vieillot, 1817)	0.02	0.02	0							
*Heterospizias meridionalis* (Latham, 1790)	0.2	0.2	0				0.03	0.03	0	Al
*Geranoaetus melanoleucus* (Vieillot, 1819)	0.17	0.17	0	0.05	0.05	0	0.06	0.06	0	Al
*Geranospiza caerulescens* (Vieillot, 1817)	0.1	0.1	0							–
Falconiformes										
Falconidae										
*Caracara plancus* (Miller, 1777)	0.27	0.27	0	0.1	0.1	0	0.04	0.04	0	Al, Me
*Herpetotheres cachinnans* (Linnaeus, 1758)	0.23	0.23	0	0.1	0.1	0	0.02	0.02	0	Cr
*Falco sparverius* Linnaeus, 1758	0.02	0.02	0							–
Cariamiformes										
Cariamidae										
*Cariama cristata* (Linnaeus, 1766)	0.36	0.34	0.02	0.1	0.1	0	0.02	0.02	0	Al, Cr
Coraciformes										
Alcedinidae										
*Chloroceryle amazona* (Latham, 1790)	0.02	0.02	0	0.05	0.05	0				Cr
Caprimulgiformes										
Caprimulgidae										
*Chordeiles acutipennis* (Hermann, 1783)	0.28	0.28	0				0.02	0.02	0	Al
Columbiformes										
Columbidae										
*Columbina minuta* (Linnaeus, 1766)	0.14	0.12	0.02				0.94	0.82	0.12	Al, Cr
*Columbina picui* (Temminck, 1813)	0.84	0.65	0.19	0.8	0.65	0.15	1.01	0.86	0.15	Al, Cr, Me
*Columbina squammata* (Lesson, 1831)	0.37	0.31	0.06	0.45	0.4	0.05	0.16	0.16	0	Al, Cr,
*Columbina talpacoti* (Temminck, 1811)	0.84	0.64	0.2	0.7	0.5	0.2	0.97	0.81	0.16	Al, Cr, Me
*Patagioenas picazuro* (Temminck, 1813)	0.48	0.42	0.06	0.25	0.25	0	0.04	0.04	0	Al, Cr
*Leptotila verreauxi* Bonaparte, 1855	0.29	0.29	0	0.3	0.3	0	0.74	0.62	0.12	Al, Cr, Me
*Claravis pretiosa* (Ferrari-Perez, 1886)				0.15	0.1	0.05	0.37	0.3	0.07	Al, Cr
*Zenaida auriculata* (Des Murs, 1847)	0.38	0.3	0.08	0.3	0.3	0	0.24	0.22	0.02	Al, Cr
Cathartiformes										
Cathartidae										
*Coragyps atratus* (Bechstein, 1793)	0.33	0.33	0	0.1	0.1	0	0.02	0.02	0	Me
*Cathartes aura* (Linnaeus, 1758)	0.02	0.02	0							Cr
Apodiformes										
Trochilidae										
*Chlorostilbon lucidus* (Shaw, 1812)	0.2	0.2	0	0.2	0.2	0	0.12	0.12	0	–
*Chrysolampis mosquitus* (Linnaeus, 1758)							0.02	0.02	0	–
*Eupetomena macroura* (Gmelin, 1788)	0.12	0.12	0				0.04	0.04	0	–
Cuculiformes										
Cuculidae										
*Guira guira* (Gmelin, 1788)	0.54	0.52	0.02	0.5	0.5	0	0.11	0.11	0	Al, Cr
*Crotophaga ani* Linnaeus, 1758	0.54	0.54	0	0.65	0.65	0	0.09	0.09	0	Al, Cr
*Crotophaga major* Gmelin, 1788	0.04	0.04	0				0.03	0.03	0	–
*Coccyzus melacoryphus* Vieillot, 1817	0.08	0.08	0							Cr
*Tapera naevia* (Linnaeus, 1766)	0.04	0.04	0							–
Charadriformes										
Jacanidae										
*Jacana jacana* (Linnaeus, 1766)	0.08	0.08	0							Al
Charadriidae										
*Vanellus chilensis* (Molina, 1782)	0.46	0.46	0	0.15	0.15	0				Al
Tinamiformes										
Tinamidae										
*Nothura boraquira* (Spix, 1825)	0.6	0.46	0.14							Al, Cr, Me
*Nothura spp*				0.35	0.35	0				Al, Cr
*Crypturellus parvirostris* (Wagler, 1827)							1.09	0.9	0.19	Al, Cr
*Crypturellus tataupa* (Temminck, 1815)	0.22	0.2	0.02	0.25	0.25	0	0.75	0.61	0.14	Al, Cr, Me
*Rhynchotus rufescens* (Temminck, 1815)							0.9	0.73	0.17	Al, Cr, Me
*Nothura maculosa* (Temminck, 1815)	0.02	0.02	0							–
Nyctibiiformes										
Nyctibiidae										
*Nyctibius griseus* (Gmelin, 1789)	0.1	0.02	0.08				0.02	0.02	0	Me
Galbuliformes										
Bucconidae										
*Nystalus maculatus* (Gmelin, 1788)	0.25	0.25	0	0.1	0.1	0	0.01	0.01	0	Al
Anseriformes										
Anatidae										
*Sarkidiornis sylvicola* Ihering & Ihering, 1907	0.31	0.31	0							Al
*Dendrocygna viduata* (Linnaeus, 1766)	0.21	0.21	0							Al
Piciformes										
Picidae										
*Veniliornis passerinus* (Linnaeus, 1766)	0.16	0.16	0	0.1	0.1	0	0.01	0.01	0	Al

Use: *Al* food, *Cr* rearing, *Me* traditional medicine

Birds of the Thraupidae and Icteridae families have been demonstrated in several studies in Brazil [12–14, 21, 22, 27–29], in which they were mainly reported as pets since they have very attractive colours, as well as a beautiful and pleasant song, according to locals. As for the Columbidae and Tinamidae families, because they include species with a higher body mass [14, 29, 30], they are one of the interviewees’ most preferred species for food; this was observed by some researchers in other regions of Brazil and is recognised and mentioned in the literature as an important food resource for the population of the northeastern semi-arid region of Brazil [21, 30, 31]. It is noteworthy that this study, as well as those carried out by Alves et al. [12] and Fernandes-Ferreira et al. [21], was conducted in rural communities, where wild birds are mainly used for food and captive breeding.

Several studies identify the value of the Emberizidae family in the context of birds used in Brazil [12, 13, 21]. However, the Brazilian Ornithological Record Committee [2] has recently relocated the Brazilian Emberizidae species to the Thraupidae family, which explains the high number of species from this family that were recorded in the present research.

Regarding the Accipitridae family, species are generally killed for retaliation purposes [32, 33]. These birds have had to change their hunting habits in some regions of Caatinga due to habitat loss and fragmentation, including adding more domestic animals in their diet, thereby generating conflicts with the human populations living in these areas. One of the causes of this change is the destruction of vegetation, which reduces hunting territory and the frequency with which prey is found [32, 33]. However, in the present study, after slaughter, these species are used as food.

The UVs ranged from 0.05 to 0.8 (UV_g_), 0.05 to 0.8 (UV_p_), and 0 to 0.2 (UV_c_) in the municipality of Lagoa. In São Mamede, these values ranged from 0.02 to 1.0 (UV_g_), 0.02 to 0.94 (UV_p_), and 0 to 0.19 (UV_c_). As for Solânea, the UVs ranged from 0.01 to 1.15 (UV_g_), 0.01 to 1.02 (UV_p_), and 0 to 0.21 (UV_c_) (Table 1). Thus, the UV_c_ were basically the same in all the studied areas. In terms of their UV_g_, *P. dominicana*, *Columbina picui* (both with 0.8), and *Columbina talpacoti* (0.7) in Lagoa; *P. dominicana* (1.0), *C. picui*, and *C. talpacoti* (both with 0.84) in São Mamede; and *S. albogularis* (1.15), *Colorhamphus parvirostris*, and *P. dominicana* (both with 1.09) in Solânea stood out because they had the highest UV. With regard to their UV_p_, *P. dominicana* (0.8), *C. picui*, and *C. talpacoti* (both with 0.65) in Lagoa; *P. dominicana* (0.94), *I. jamacaii* (0.88), and *C. cyanopogon* (0.79) in São Mamede; and *P. dominicana* (1.02), *S. albogularis* (0.94), and *C. parvirostris* (0.9) in Solânea were the most prominent species. As for their UV_c_, *C. talpacoti* (0.2) and *C. picui* (0.15) in Lagoa, *C. picui* (0.19) and *Nothura boraquira* (0.14) in São Mamede, and *S. albogularis* (0.21), *C. parvirostris* (0.19), and *Sporophila lineola* (0.18) in Solânea were the most prominent species.

Species with high use values (UV_c_ and UV_p_), such as *S. albogularis*, *C. talpacoti*, *C. picui*, *S. lineola*, *P. dominicana*, *I. jamacaii*, and *C. parvirostris*, have been recorded in several studies involving local populations in Brazil [12–14, 21, 34]. There is a need for in-depth studies of the distribution and abundance of these species, as well as of the impacts caused by the indiscriminate removal of these species from their natural environment. Another important finding is that, independent of the studied localities, there was a great valuation of some species, such as *P. dominicana*, *S. albogularis*, and the species of the genus *Columbidae* sp., which suggests a possible standard of use for such species.

From the data, we observed variation in the ranking of the species with different UVs, which indicates that the species that are actually used (UV_c_) may be under a high pressure of use and overexploitation. With respect to the UV_p_, these species also require attention because they are potential targets for use and may be incorporated into regular use if the species that are actually used become extinct [20, 35, 36]. This distinction also identifies that several factors (i.e. purpose of use, local availability, abundance, and cultural factors) are associated with the choice and use of wild birds [13, 21, 31, 34].

The majority of the species had UV_g_ lower than one (1); nevertheless, four species had higher values, reflecting the local importance of these animals in the studied areas. Use value [37–39] is the most widely used technique among different researchers, and its results are generally interpreted as the possible use pressure on the species [40–43]. However, it does not distinguish between current use (effective) and potential use; therefore, the application of the UV_g_ becomes unreliable for allocating a high number of potential uses in the analysis. This distinction between knowledge and effective use needs to be addressed in future studies for the development of conservation and management initiatives based on accurate information, for species that are actually under pressure [20, 34, 35, 44].

Such information shows that the application of the UV, from the differentiated formulas, results in more cohesive and in-depth diagnoses with respect to the cultural importance of the mentioned species [20, 35, 36]. Soares et al. [34] noted that the differentiation between current use and potential use is important for conservation studies since it can better explain the effective use of natural resources, in addition to being significantly different from the general use value.

Rearing (pets) was the use category with the highest number of species (Table 1), with 22, 41, and 47 species recorded in Lagoa, São Mamede, and Solânea, respectively. These data corroborate several studies [12–14, 21–23, 27, 45–47] involving bird breeders, free-market traders, wild bird markets, and rural community residents, proving that this activity is widespread in all regions of the world. Alves et al. [47] estimate that 400 wild bird species are illegally traded in Brazil. This richness of species can also be observed in Southeast Asia (291 species) and Africa (354 species) [48, 49]. Birdsong, followed by colour and an affection for birds, is the most important and decisive factor for choosing a species for rearing in captivity [12, 13, 21, 23, 46].

*P. dominicana*, *C. picui*, *C. talpacoti*, *I. jamacaii*, *S. albogularis*, and *S. lineola* were the species that were most frequently cited for captive breeding in the three communities. They are used due to their song and plumage beauty, which, according to Sick [1], are the main factors that motivate captive breeding. In several regions, the practice of keeping birds in cages is so widespread that people still use ornamental cages, or even cages containing fake birds (i.e. ornaments or artificial birds) [12, 13].

Thraupidae was the most cited family in the pet category (10 species) (Table 1). *S. albogularis* had the highest current use value (UV_c_ = 0.21), suggesting that it is possibly under the pressure of use. Species of different families are bred in several regions worldwide [12–14, 21, 22, 27, 45, 50]. However, because they have a large repertoire of vocalisations, species belonging to the Thraupidae family are more frequently used and stand out in comparison with the others; this family is one of the most confiscated wild bird families in the illegal wildlife trade in many parts of the world [27, 28, 45, 46, 50]. The wide appreciation and use of species of the *Sporophila* genus has been demonstrated in several studies in Brazil [12, 21, 22, 27, 46]. Due to their beauty and an appreciation for their song, species in this genus have become very widespread in the region.

The use of wild birds as a food resource was the most cited category of use in Solânea (52 species), with *R. rufensces*, *Crypturellus tataupa*, and *C. picui* being the most prominent species. In Lagoa and São Mamede, food was the category with the second highest number of species (16 and 29 species, respectively), with *C. talpacoti*, *C. picui*, *Zenaida auriculata*, and *N. boraquira* standing out. These species are used in both communities because of their abundance, the ease with which they are captured (Columbidae), and their body size (Tinamidae).

The consumption of wild birds has occurred in some regions of northeast Brazil since 1600 [29]. Due to high diversity, the number of individuals, and their high protein value, birds became an important food resource for the population of the northeastern semi-arid region of Brazil [14, 22, 30, 31] and around the world [22, 51–56]. Due to the extreme seasonal changes in this ecosystem, populations developed a strong dependence on the animal resources available in the environment [16, 17]. The majority of the used species as food are of great nutritional importance for the families living in the Sertão region [14, 22, 30, 31]. Parry et al. [53] and Van Vliet et al. [54] showed that the consumption of meat from wild animals goes beyond the local ambit, making this meat product present in several wild animal markets; this led to an increase in the capture and slaughter of these animals, causing severe impacts on the populations of the involved species.

Species belonging to the Columbidae and Tinamidae families represent the most important Brazilian used as food birds, since they provide the rural populations with some of the proteins that are indispensable for survival [1, 30, 31]. Robinson and Redford [55] explained that species of these families are among those that are most used for subsistence hunting in the Neotropical region. Bezerra et al. [30] and Endo et al. [56] stated that these species have a significant body mass in common, which is a decisive characteristic when hunting for food purposes. However, Mendonça et al. [31] showed that, although species of the Tinamidae family have a larger body mass, compared to the Columbidae species, the availability and abundance of columbiform birds makes their family highly valuable for food purposes. However, if there are no large-sized species, such as those of the Tinamidae family, the informants explained that typical and easily available species are more widely used, as reported by Bezerra et al. [30] and Mendonça et al. [31]. Alves et al. [12] noted that Columbidae species are also widely used in captive breeding (pets), corroborating the present study, in which the use of *C. talpacoti* and *C. picui* was recorded in Lagoa and São Mamede. According to Marini et al. [57], many species of the Columbidae family are adapted to disturbed and/or anthropogenised habitats and reproduce throughout the year, which may explain the wide availability and diversity of use of the species, as mentioned in the present study.

Some species are used in traditional medicine (8 in São Mamede, 5 in Solânea, and 1 in Lagoa) (Table 2), corroborating other studies in Brazil and worldwide [17, 49, 58–62]. These studies noted that birds are frequently used for medicinal purposes in local cultures, either through the use of body parts or the whole bird. For example, *C. cyanopogon* (white-naped Jay) is raised in captivity with the intention of absorbing the diseases (asthma and bronchitis) of sick people, according to the informants. This bird is kept in captivity until the patient is cured, as reported in other studies in the Caatinga region [17, 21, 60, 61].

**Table 2:** Species used in traditional medicine in the municipalities of Solânea, Lagoa, and São Mamede in Paraíba State, Northeast Brazil

Species	Used parts	How to use	Treated diseases
*Nothura boraquira* (Spix, 1825)	Feathers	Boil water and add a powder made of feather and drink twice a day.	Snake bite/bleeding
*Columbina picui* (Temminck, 1813)	Meat	Remove the meat from the gizzard, bake without salt and eat.	Lack of appetite/gastritis
*Columbina talpacoti* (Temminck, 1811)	Meat	Remove the meat from the gizzard, bake without salt, and eat.	Falta de apetite/gastrite
*Cyanocorax cyanopogon* (Wied, 1821)	Whole bird	When the sick person feeds, leaves a rest, and gives the bird to eat there, the disease passes to the bird and it dies and the person is cured.	Asthma/bronchitis
*Leptotila verreauxi* Bonaparte, 1855	Skin	Remove the meat from the gizzard, toast, and apply to the affected site	Vilidia
*Paroaria dominicana* (Linnaeus, 1758)	Whole bird	Kill the bird and place it on top of the affected spot.	Panarisso
*Crypturellus tataupa* (Temminck, 1815)	Whole bird	Kill the bird bake without salt and eat	Pregnancy sickness
*Caracara plancus* (Miller, 1777)	Meat	Eat the “pirão” (preparation made with the meat of the bird)	Weakness
*Rhynchotus rufescens* (Temminck, 1815)	Feathers	Burn the feather and make a tea	Snake bite/shortness of breath
*Coragyps atratus* (Bechstein, 1793)	Feathers/foot	Burn the feather and make a tea /burn and toast the foot, add boiled water, and give the person a drink without her knowing	Tiredness/alcoholic
*Fluvicola nengeta* (Linnaeus, 1766)	Whole bird	Burn the whole bird until it turns a powder and make the tea with the powder and take.	Asthma
*Nyctibius griseus* (Gmelin, 1789)	Feathers	Burn the feather and make a tea.	Tiredness and shortness of breath

*Rhynchotus rufescens* and *N. boraquira* were the most cited species (3 and 6 citations). The medicinal species recorded in the present study corroborate with the results of other studies [17, 58, 60, 61]. The medicinal use of *Nyctibius griseus* was recorded for the first time in our study. Its feathers are burned and used to make tea for people with fatigue or asthma. Costa-Neto and Alves [61] recorded 47 species of wild birds that were used for medicinal purposes in Brazil and Bezerra et al. [60] recorded six new species that were used for this same purpose. These data indicate that at least 54 species are used for medicinal purposes in Brazil, corresponding to 45% of the wild bird species used in Latin America [17].

In the present study, the use of body parts of *Coragyps atratus*, such as the feet and toasted feathers to make tea for the treatment of fatigue and alcoholism (to stop drinking), was recorded. The use of parts of *C. atratus* was reported in other studies, for several therapeutic purposes, such as the treatment of asthma [17, 21, 59, 60, 62], earache [21], alcoholism [17, 60], and alternative therapies in the treatment of cancer [59]. However, Bezerra et al. [60] emphasised that, in the case of alcoholism treatment, the patient cannot know he/she is ingesting the traditional preparation of toasted feathers.

According to Alves et al. [58] and Feng et al. [63], several factors must be considered in future studies aimed to investigate the use of animals in traditional medicine, such as the suffering caused by the capture processes and the preparation of treatments, as well as the risks to those who consume such medicines. Alves et al. [58] and Costa-Neto and Alves [61] state that the therapeutic purpose of faunal resources should be a topic of discussion in conservation biology, public health, the sustainable management of natural resources, biological prospecting, and patent law. Bezerra et al. [60] emphasise that there is no available information on the use and applicability of remedies formed from wild birds, as well as on their pharmacological efficacy; this highlights the need for in-depth studies on this subject.

Several studies [21, 23, 27, 28, 31, 45, 51–56] have highlighted that, despite the different forms of use attributed to birds by contemporary societies, food and illegal trade are the main purposes that lead to the slaughter and capture of wild birds around the world and are among the main factors responsible for declining populations [23, 27, 55, 64]. The use of biodiversity negatively affects ecosystems, causing a large population decline of several species around the world [64–66]. On the other hand, the use of these resources plays a fundamental role in the life of the people living in the Sertão region of northeast Brazil, especially during long drought periods when the growth of agricultural crops becomes difficult and domestic animals are decimated by hunger and thirst [21, 31].

Although the use of wild fauna is a common practice in northeastern Brazil, in-depth studies on this subject are scarce and there is a lack of information about which species are most used by the local populations, and for what purposes, as well as the implications for the conservation of the exploited birds. Thus, the data presented in this study show that integrating cultural values with scientific analysis and information may elucidate ways of mitigating the impact of anthropogenic pressures on wild bird populations, as well as contribute to future efforts for the conservation of the used species.

### Conservation implications

According to the IUCN [26], Brazil is the country with the most endangered avifauna. A total of 189 bird species are on the global list of endangered species [26], and 160 are on the national list [11]. Excessive capture represents the main threat to Brazilian avifauna [12, 27, 28], followed by other factors, such as habitat loss and fragmentation, the introduction of exotic species, pollution, changes in the dynamics of native species, and persecution [3, 67]. Despite being illegal, hunt activities are closely associated with cultural issues [12, 21, 31, 53, 54, 56, 68] and most used species are of great nutritional importance to the people from the Sertão region [12, 14, 30, 31]. Most of the population of the semi-arid region of Brazil lives in poor socioeconomic conditions [17]; therefore, according to Santos and Costa-Neto [29], it is difficult to discuss biodiversity conservation when a considerable part of the population must resort to the wild fauna as a subsistence alternative.

Birds are the animal group under the strongest anthropogenic pressure in the northeastern semi-arid region, both economically (e.g. songbirds) and from the point of view of subsistence trophic connection [12–14, 21, 30, 31, 45]. Several studies [64–66, 68, 69] have noted that the over-exploitation of wild birds causes imbalances in food, involving other groups and having serious environmental consequences. According to Harrison [66] and Reyers et al. [69], most of the used species are pollinators and seed dispersers, so the hunting of wild birds in the Caatinga region has important conservation implications. The species used by local human populations are listed as endangered animals [11, 26], such as *Spinus yarrellii*, which was recorded in this study and is categorised as vulnerable according to the Ministry of Environment (MMA) [11].

Marini and Garcia [3] claim that, in Brazil, few states have compiled lists of endangered species, which are essential for publicising information on fauna and influencing and directing conservation measures. The rapid development of lists at the regional level verifies the availability of wild bird populations, indicating species that are under hunted pressure.

Conflicts between wildlife and humans consist of a widespread conservation issue that is a growing interest for conservationists [32, 33, 65, 66]. In this perspective, ethno-ornithological studies are good interpretive tools through which to study the interactions between humans and birds in any given region [13, 15]; they contribute to ensuring that wild fauna is properly valued, not only from an ecological point of view, but also economically and socially, in addition to providing subsidies for the implementation of environmental management and species conservation [70].

According to Baruch-Moro et al. [70], both traditional knowledge and scientific information should be combined to solve practical problems, organising the hunt activities in a sustainable way by including community participation in species conservation.

## Final considerations

The residents of the studied communities have a relevant knowledge of the avifauna, as well as of their forms of use. The use of the different UV calculations provided an accurate observation of the actually used species, that is, it identified the birds that are under a high pressure of use.

The main form of avifauna use in the three studied communities was related to breeding, followed by food. This fact can be demonstrated by observing the highest UVs of the species listed by each informant.

The present data show the importance of conducting studies involving traditional knowledge, since they highlight relevant information that can be used in the development of management and conservation plans; this makes the sustainable use of resources relating to the fauna in each community possible, especially in regions of the Caatinga, which has suffered from strong anthropogenic actions.

## Abbreviations

CAAE: Certificate of Presentation for Ethical Consideration
CBRO: Brazilian Ornithological Records Committee
CEP: Research Ethics Committee
HULW: Lauro Wanderley University Hospital
IUCN: International Union for Conservation of Nature
UV: Use value index
UV_a_: Use value current
UV_p_: Use value potential

## References

[1] Sick H (1997). Ornitologia brasileira.

[2] Piacentini VQ, Aleixo A, Agne CE, Maurício GN, Pacheco JF, Bravo GA, Brito GRR, Naka LN, Olmos F, Posso S, Silveira LF, Betini GS, Eduardo Carrano E, Franz I, Lees AC, Lima LM, Pioli D, Schunck F, Amaral FR, Bencke GA, Cohn-Haft M, Figueiredo LFA, Straube FC, Evaldo Cesari E (2015). Annotated checklist of the birds of Brazil by the Brazilian Ornithological Records Committee / Lista comentada das aves do Brasil pelo Comitê Brasileiro de Registros Ornitológicos. Revista Brasileira de Ornitologia.

[3] Marini MA, Garcia FI (2005). Bird conservation in Brazil. Conserv Biol.

[4] Del Hoyo J, Elliott A, Chritie DA (2014). Handbook of the birds of the world - volume 16. Linx Edicions.

[5] Ab’saber AN (1973). A organização natural das paisagens inter e subtropicais brasileiras. Geomorfologia.

[6] de Albuquerque Ulysses Paulino, de Lima Araújo Elcida, El-Deir Ana Carla Asfora, de Lima André Luiz Alves, Souto Antonio, Bezerra Bruna Martins, Ferraz Elba Maria Nogueira, Maria Xavier Freire Eliza, Sampaio Everardo Valadares de Sá Barreto, Las-Casas Flor Maria Guedes, de Moura Geraldo Jorge Barbosa, Pereira Glauco Alves, de Melo Joabe Gomes, Alves Ramos Marcelo, Rodal Maria Jesus Nogueira, Schiel Nicola, de Lyra-Neves Rachel Maria, Alves Rômulo Romeu Nóbrega, de Azevedo-Júnior Severino Mendes, Telino Júnior Wallace Rodrigues, Severi William (2012). Caatinga Revisited: Ecology and Conservation of an Important Seasonal Dry Forest. The Scientific World Journal.

[7] Muller P (1973). Dispersal centers of terrestrial vertebrates in the neotropical. Biogeographica.

[8] Haffer J. Avian zoogeography of the Neotropical lowland. Ornithol Monogr. 1985;(36):113–46.

[9] Rizzini CT (1997). Tratado de Fitogeografia do Brasil. 2° Edição.

[10] Silva JMCV, Leal I, Tabarelli M. Caatinga the largest tropical forest region in South America: 1. Ed. Berlim: Springer. 2017. p. 487.

[11] Silveira LF, Straube FC, Machado AB, Drummond GM, Paglia AP (2008). Aves ameaçadas de extinção no Brasil. Livro vermelho da fauna brasileira ameaçada de extinção. 1ª ed.

[12] Alves RRN, Nogueira EEG, Araujo HFP, Brooks SE (2010). Bird-keeping in the Caatinga, NE Brasil. Hum Ecol.

[13] Alves RRN, Leite RCL, Souto WMS, Bezerra DMM, Loures-Ribeiro A (2013). Ethno-ornithology and conservation of wild birds in the semi-arid Caatinga of northeastern Brazil. J Ethnobiol Ethnomed.

[14] Teixeira PHR, Thel TN, Ferreira JMR, Azevedo-Jr SM, Telino-Jr WR, Lyra-Neves RM (2014). Local knowledge and exploitation of the avian fauna by a rural community in the semi-arid zone of northeastern Brazil. J Ethnobiol Ethnomed.

[15] Vásquez-Dávila MA (2014). Aves, Personas y Culturas Estudios de Etno-ornitologia 1.

[16] Sampaio Y, Batista JEM, Silva JMC, Tabarelli M, Fonseca MT, Lins LV (2004). Desenvolvimento Regional E Pressões Antrópicas No Bioma Caatinga. Biodiversidade da Caatinga: Áreas a ações prioritárias para a Conservação.

[17] Alves RRN, Alves HN (2011). The faunal drugstore: animal-based remedies used in traditional medicines in Latin America. J Ethnobiol Ethnomed.

[18] IBGE, 2013. Disponível em: https://cidades.ibge.gov.br/. Acesso em: 11 de outubro de 2018.

[19] Albuquerque UP, Cunha LUFC, Lucena RFP, Alves RRN (2014). Methods and techniques in ethnobiology and ethnoecology. 1. Ed.

[20] Lucena RFP, Medeiros PM, Araújo E, Alves AGC, Albuquerque UP (2012). The ecological apparency hypothesis and the importance of useful plants in rural communities from Northeastern Brazil: an assessment based on use value. J Environ Manag.

[21] Fernandes-Ferreira H, Mendonça SU, Ferreira CAFS, Alves RRN (2012). Hunting, use and conservation of birds in the Northeast Brazil. Biodivers Conserv.

[22] Loss ATG, Costa-Neto EM, Flores FM (2014). Aves silvestres utilizadas como recurso trófico pelos moradores do povoado de Pedra Branca, Santa Teresinha.

[23] Liang W, Cai Y, Yang CC (2011). Extreme levels of hunting of birds in a remote village of Hainan Island, China. Bird Conservation International..

[24] Sigrist T. The Avis Brasilis field guide to the birds of Brazil. Plates and maps. Vinhedo. Avis Brasilis. 2009.

[25] Lyra-Neves RM, Telino-Junior WR (2010). As aves da Fazenda Tamanduá. Avis Brasilis.

[26] BirdLife International. 2016. Spinus yarrellii. The IUCN Red List of Threatened Species 2016: http://dx.doi.org/10.2305/IUCN.UK.2016-3.RLTS.T22720368A94666662.en. Downloaded on 31 March 2017.

[27] Regueira Rodrigo Farias Silva, Bernard Enrico (2012). Wildlife sinks: Quantifying the impact of illegal bird trade in street markets in Brazil. Biological Conservation.

[28] Nascimento CAR, Czaban RE, Alves RRN (2015). Trends in illegal trade of wild birds in Amazonas state, Brazil. Tropical Conservation Science..

[29] Santos IB, Costa-Neto EM (2007). Estudo etnoornitológico em uma região do Semiárido do estado da Bahia, Brasil. Sitientibus série Ciências Biológicas.

[30] Bezerra DMM, Araujo HFP, Alves RRN (2011). Avifauna silvestre como recurso alimentar em áreas de semiárido no estado do Rio Grande do Norte, Brasil. Sitientibus – Série Ciências Biológicas.

[31] Mendonça Lívia E. T., Vasconcellos Alexandre, Souto Caroline M., Oliveira Tacyana P. R., Alves Rômulo R. N. (2015). Bushmeat consumption and its implications for wildlife conservation in the semi-arid region of Brazil. Regional Environmental Change.

[32] Mendonça LET, Souto CM, Andrelino LL, Souto MSW, Vieira WLS, Alves RRN (2011). Conflitos entre pessoas e animais silvestres no Semiárido paraibano e suas implicações para conservação. Sitientibus Série Ciências Biológicas.

[33] Moleón Marcos, Sánchez-Zapata José A., Gil-Sánchez José M., Barea-Azcón José M., Ballesteros-Duperón Elena, Virgós Emilio (2011). Laying the Foundations for a Human-Predator Conflict Solution: Assessing the Impact of Bonelli's Eagle on Rabbits and Partridges. PLoS ONE.

[34] Soares VMS, Soares HKL, Lucena RFP, Barboza RRD (2018). Conhecimento, uso alimentar e conservação da avifauna cinegética: Estudo de caso no município de Patos, Paraíba. Inteciencia.

[35] Ribeiro JES, Carvalho TKN, Ribeiro JPO, Guerra NM, Silva N, Pedrosa KM, Alves CAB, Souza-Júnior SP, Souto JS, Nunes AT, Lima JRF, Oliveira RS, Lucena RFP (2014). Ecological apparency hypothesis and availability of useful plants: testing different use values. Acta botanica Brasilica.

[36] Guerra NM, Ribeiro JES, Carvalho TKN, Pedrosa KM, Felix LP, Lucena RFP. Usos locais de espécies vegetais nativas em uma comunidade rural no Semiárido Nordestino (São Mamede, Paraíba, Brasil). Biofar Volume especial. 2012:186–212.

[37] Phillips O, Gentry AH (1993). The useful plants of Tambopata, Peru: I. statistical hypothesis tests with a new quantitative technique. Econ Bot.

[38] Phillips O, Gentry AH (1993). The useful plants of Tambopata, Peru II. Additional hypothesis testing in quantitative ethnobotany. Econ Bot.

[39] Rossato SC, Leitão-Filho HF, Begossi A (1999). Ethnobotany of Caiçaras of the Atlantic Forest coast (Brazil). Econ Bot.

[40] Albuquerque UP, Lucena RFP, Monteiro JMM, Florentino ATN, Almeida CFR (2006). Evaluating two quantitative ethnobotanical techniques. Ethnobotanical Research and Applications.

[41] Melo RS, Silva OC, Souto A, Alves RRN, Shiel N (2014). The role of mammals in local communities living in conservation areas in the Northeast of Brazil: an ethnozoological approach. Tropical Conservation Science.

[42] Silva VA, Nascimento VT, Soldati GT, Medeiros MTF, Albuquerque UP, Albuquerque UP, LVFC C, RFP L, RRN A (2014). Techniques for analysis of quantitative ethnobiological data: use of indices. Methods and techniques in ethnobiology and ethnoecology.

[43] Oliveira WSL, Lopes SF, Alves RRN (2018). Understanding the motivations for keeping wild birds in the semi-arid region of Brazil. J Ethnobiol Ethnomed.

[44] Nunes EM, Guerra NM, Arévalo-Marín E, Alves CAB, Nascimento VT, Cruz DD, Ladio AH, Silva SM, Oliveira RS, Lucena RFP (2018). Local botanical knowledge of native food plants in the semiarid region of Brazil. J Ethnobiol Ethnomed.

[45] Daut EF, Brights DJ, Mendonza AP, Puhakka L, Peterson MJ (2015). Illegal domestic bird trade and the role of expert quotas in Peru. J Nat Conserv.

[46] Rocha MSP, Cavalcanti PCM, Sousa RL, Alves RRN (2006). Aspectos da comercialização ilegal de aves nas feiras livres de Campina Grande, Paraíba, Brasil. Revista de Biologia e Ciências da Terra.

[47] NÓBREGA ALVES RÔMULO ROMEU, DE FARIAS LIMA JOSÉ RIBAMAR, ARAUJO HELDER FARIAS P. (2012). The live bird trade in Brazil and its conservation implications: an overview. Bird Conservation International.

[48] Nijman V (2010). An overview of international wildlife trade from Southeast Asia. Biodivers Conserv.

[49] Williams VL, Cunningham AB, Kemp A, Bruyns RK (2014). Risk to birds traded for African traditional medicine: a quantitative assessment. PLoS One.

[50] Roldán-Clara B, Toledo VM, Espejel I (2017). The use of birds as pets in Mexico. J Ethnobiol Ethnomed.

[51] Drury R (2009). Reducing urban demand for wild animals in Vietnam: examining the potential of wildlife farming as a conservation tool. Conserv Lett.

[52] Nasi R, Tabe A, Van Vliet N (2011). Empty forests, empty stomachs? Bushmeat and livelihoods in the Congo and Amazon Basins. Int For Rev.

[53] Parry L, Barlow J, Pereira H (2014). Wildlife harvesting and consumption in Amazonia’s urbanized wilderness. Conserv Lett.

[54] Van Vliet N, Quiceno-Mesa MP, Cruz-Antia D, Tellez L, Martins C, Haiden E, Oliveira MR, Adams C, Morsello C, Valencia L. From fish and bushmeat to chicken nuggets: the nutrition transition in a continuum from rural to urban settings in the Tri frontier Amazon region. Ethnobiology and Conservation. 2015. 10.15451/ec2015-7-4.6-1-12.

[55] Robinson JG, Redford KH (1994). Measuring the sustainability of hunting in tropical forests. Oryx.

[56] Endo W, Peres CA, Salas E, Mori S, Sanches-Vega JL, Shepard GH, Pacheco V, Yu DW. Game vertebrate densities in hunted and non-hunted forest sites in Manu National Park, Peru. Biotropica. 2010; doi.org/10.1111/j.1744-7429.2009.00546.x.

[57] Marini MA, Borges FJ, Lopes LE, França L, Duca L, Paiva LV, Manica LT, Gressler DT, Heming NM (2010). Breeding biology of Columbidae in central Brazil. Ornitologia Neotropical.

[58] Alves RRN, Rosa IL, Santana GG (2007). The role of animal-derived remedies as complementary medicine in Brazil. Bioscience.

[59] Sánchez-Pedraza R, Gamba-Rincón MR, González-Rangel AL (2012). Use of black vulture (*Coragyps atratus*) in complementary and alternative therapies for cancer in Colombia: a qualitative study. J Ethnobiol Ethnomed.

[60] Bezerra DMM, Araújo HFP, Alves AGC, Alves RRN (2013). Birds and people in semiarid northeastern Brazil: symbolic and medicinal relationships. J Ethnobiol Ethnomed.

[61] Costa-Neto EM, Alves RRN (2010). Zooterapia: os animais na medicina popular brasileira. Recife: Nupeea.

[62] Vargas-Clavijo M, Costa-Neto EM. Los limpadores de los cielos: factos y folclor de los zopilotes, aves duenas del imaginario latinoamericano. Feira de Santana. 2008:207.

[63] Feng Y, Siu K, Wang N, Kwan-Ming N, Tsao S, Nagamatsu T, Tong Y (2009). Bear bile: dilemma of traditional medicinal use and animal protection. J Ethnobiol Ethnomed.

[64] Redford KH (1992). The empty forest. Bioscience.

[65] Dickman AJ (2010). Complexities of conflict: the importance of considering social factors for affectively resolving human wildlife conflict. Anim Conserv.

[66] Harrison RD (2011). Empting the forest: hunting and the extirpation of wildlife from tropical nature reserves. Bioscience.

[67] Machado N, Loyola RDA (2013). Comprehensive quantitative assessments of bird extinction in Brazil. PLoS One.

[68] Hill K, Padwe J, Bejyvagi C, Bepurange A, Sakugi F, Tykuarangi R, Tykuarangi T (1997). Impact of hunting on large vertebrates in the Mbaracayu Reserve, Paraguay. Conserv Biol.

[69] Reyers B, Pettorelli N, Katzner T, Gompper ME, Gordon IJ (2010). Animal conservation and ecosystem services: garnering the support of mightier forces. Anim Conserv.

[70] Baruch-Mordo S, Breck SW, Wilson KR, Broderick J (2011). The carrot or the stick? Evaluation of education and enforcement as management tools for human-wildlife conflicts. PLoS One.

